# Mitochondrial calcium uptake in organ physiology: from molecular mechanism to animal models

**DOI:** 10.1007/s00424-018-2123-2

**Published:** 2018-03-15

**Authors:** Cristina Mammucari, Anna Raffaello, Denis Vecellio Reane, Gaia Gherardi, Agnese De Mario, Rosario Rizzuto

**Affiliations:** Department of Biomedical Sciences, University of Padova, Padua, Italy

**Keywords:** Mitochondria Ca^2+^ uptake, Animal models, Heart, Skeletal muscle, Neurons, Pancreatic β cells

## Abstract

Mitochondrial Ca^2+^ is involved in heterogeneous functions, ranging from the control of metabolism and ATP production to the regulation of cell death. In addition, mitochondrial Ca^2+^ uptake contributes to cytosolic [Ca^2+^] shaping thus impinging on specific Ca^2+^-dependent events. Mitochondrial Ca^2+^ concentration is controlled by influx and efflux pathways: the former controlled by the activity of the mitochondrial Ca^2+^ uniporter (MCU), the latter by the Na^+^/Ca^2+^ exchanger (NCLX) and the H^+^/Ca^2+^ (mHCX) exchanger. The molecular identities of MCU and of NCLX have been recently unraveled, thus allowing genetic studies on their physiopathological relevance. After a general framework on the significance of mitochondrial Ca^2+^ uptake, this review discusses the structure of the MCU complex and the regulation of its activity, the importance of mitochondrial Ca^2+^ signaling in different physiological settings, and the consequences of MCU modulation on organ physiology.

## Introduction

Ca^2+^ accumulation in energized mitochondria was first described in the early 1960s [22, 110], and since then, the contribution of Ca^2+^ uptake to mitochondrial bioenergetics and cellular processes has considerably evolved. The formulation of the chemiosmotic hypothesis [66], together with the measurement of sizable internally negative membrane potentials [94], led to the concept of an energetically favorable Ca^2+^ uptake mechanism. In the 1980s, with the identification of inositol 1,4,5-trisphosphate (InsP_3_) as a soluble second messenger, which triggers the release of Ca^2+^ from the endoplasmic reticulum (ER), and the development of accurate tools for measuring Ca^2+^ concentration, the attention on mitochondrial Ca^2+^ accumulation declined. Indeed, it appeared that the affinity of mitochondria for Ca^2+^ was too low to accumulate the cation, not only in resting cytosolic Ca^2+^ concentrations ([Ca^2+^]_cyt_) (~ 0.1 μM) but also during the transient increase (2–3 μM) generated by cell stimulation [65]. This view was drastically revised when tools allowing the selective measurement of mitochondrial Ca^2+^ concentration ([Ca^2+^]_m_) in living cells were developed. By targeting the Ca^2+^-sensitive photoprotein aequorin to mitochondria, Pozzan and coworkers demonstrated that a rapid mitochondrial Ca^2+^ peak, reaching values well above those of the bulk cytosol, parallels the [Ca^2+^]_cyt_ rise evoked by cell stimulation [91]. Furthermore, the apparent discrepancy between the affinity of Ca^2+^ transporters and the high level of Ca^2+^ taken up by mitochondria was resolved by demonstrating that mitochondria, upon cell stimulation, are exposed to microdomains of high [Ca^2+^] that greatly exceed the values measured in the cytosol due to the close contacts (< 200 nm) between the mitochondria and the ER [92].

**Fig. 1:**
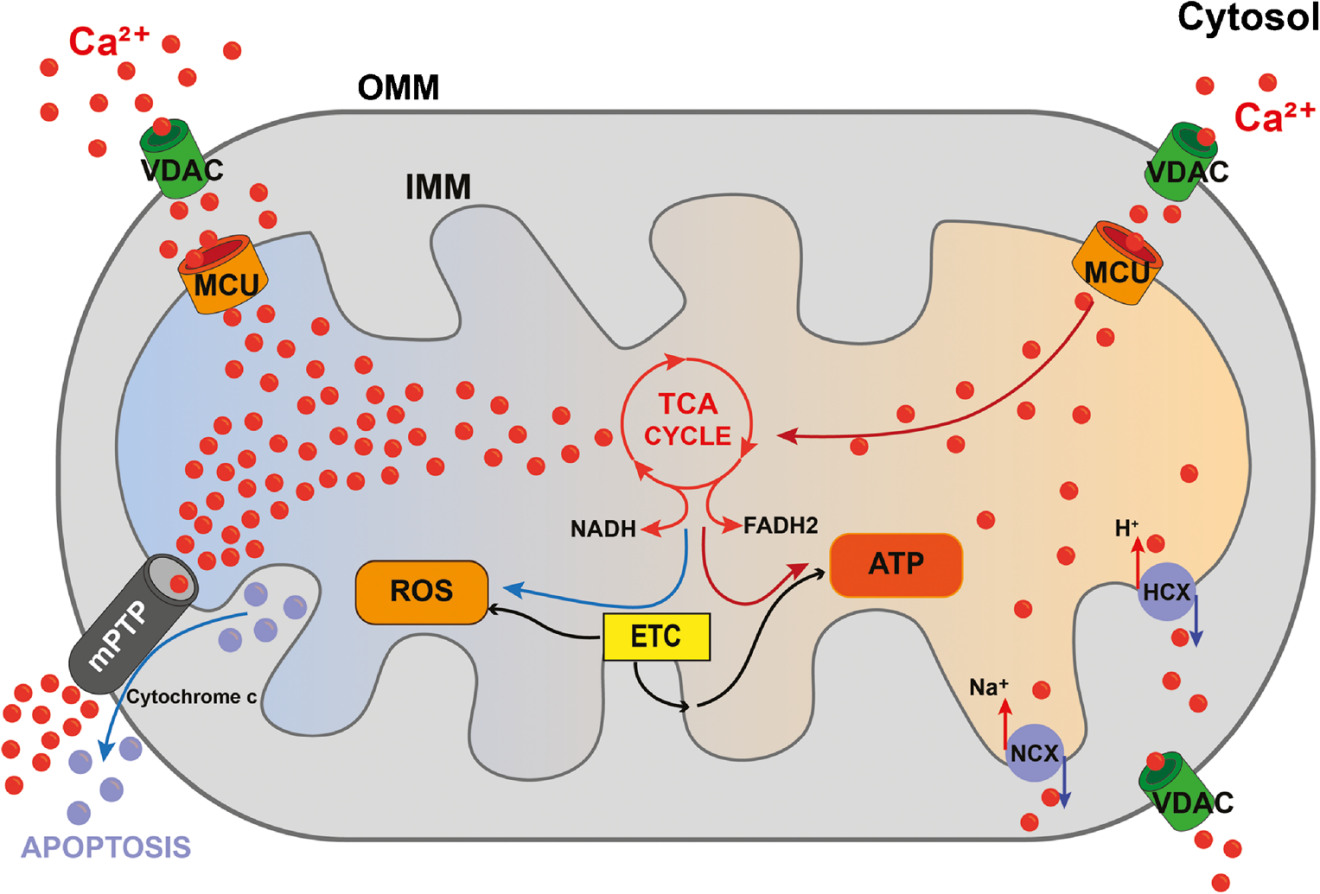
Mitochondrial Ca^2+^ homeostasis is regulated by influx and efflux mechanism and impinges on oxidative metabolism, mROS generation, and mPTP opening. Physiologically, mitochondrial Ca^2+^ uptake stimulates TCA cycle and ATP production (right-hand side), while in pathological conditions, mitochondrial Ca^2+^ overload causes the opening of the mPTP (left-hand side). mROS play either a signaling role or behave as damaging agents depending on their concentration and on the biological context

Mechanistically, to reach the mitochondrial matrix, Ca^2+^ needs to cross two lipid bilayers: the outer and the inner mitochondrial membranes (Fig. 1). The outer mitochondrial membrane (OMM) is permeable to ions and small proteins (MW < 10 kDa), thanks to the presence of a large conductance channel, the voltage-dependent anion channel (VDAC), whose permeability is controlled by ATP and other regulatory factors [18]. The inner mitochondrial membrane (IMM) is an ion-impermeable membrane, whose surface is significantly bigger than the one of OMM due to the presence of numerous invaginations called *cristae*. The huge driving force for mitochondrial Ca^2+^ entry is provided by the activity of the respiratory chain complexes that, by translocating H^+^ in the intermembrane space, leads to an electrochemical gradient (Δ*μ*_H_) that in mitochondria is mainly represented by the electrical component, generating a mitochondrial membrane potential (Δ*Ψ*_m_) of ~ 180 mV. Accordingly, treatment with an uncoupler such as *p*-[trifluoromethoxyl]-phenyl-hydrazone (FCCP), that collapses the Δ*Ψ*_m_, abolishes mitochondrial Ca^2+^ uptake. [Ca^2+^]_m_ is additionally regulated by Ca^2+^ efflux pathways through the mitochondrial Na^+^/Ca^2+^ (NCLX) and H^+^/Ca^2+^ (mHCX) exchangers [93].

The functional significance of mitochondrial Ca^2+^ accumulation started to be elucidated when it was demonstrated that mitochondrial Ca^2+^ regulates three key enzymes of mitochondrial metabolism: ketoglutarate dehydrogenase, isocitrate dehydrogenase, and pyruvate dehydrogenase phosphatase 1 (PDP1). The net effect on tricarboxylic acid (TCA) cycle activation is a boost in the synthesis of reduced OXPHOS substrates (NADH and FADH_2_), enhanced respiratory chain activity, and a subsequent increase in H^+^ pumping [93] (Fig. 1).

In addition, Ca^2+^ pulses also stimulate the adenine nucleotide transporter [63] and complex V (mitochondrial F_0_F_1_ ATP synthase) [20], harnessing the H^+^ gradient to upregulate ATP production. Finally, Ca^2+^ activates α-glycerolphosphate dehydrogenase, a component of the glycerol phosphate shuttle that supplies NAD^+^ for glycolysis [116]. Thus, an important role for mitochondrial Ca^2+^ accumulation could be inferred, i.e., a rapid upregulation of mitochondrial ATP production in stimulated cells.

The ability of the mitochondria to act as Ca^2+^ buffers impinges also on the pattern of the cytosolic Ca^2+^ signals, with different consequences depending on the arrangement of mitochondria inside cells. For example, in pancreatic acinar cells, three distinct groups of mitochondria have been identified, i.e., mitochondria located at the peripheral basal area and perigranular and perinuclear mitochondria. Each of these subsets is characterized by specific responses to cytosolic Ca^2+^ signals occurring in their close proximity [76].

The mitochondrial electron transport chain is the main cellular process that generates reactive oxygen species (ROS) in mammalian cells under both physiological and pathological conditions. ROS are derived from molecular oxygen by electron transfer reactions resulting in the formation of superoxide anion radical (O^2−^) and subsequently hydrogen peroxide (H_2_O_2_), either spontaneously or by the action of superoxide dismutases (SOD). In the presence of iron, superoxide and H_2_O_2_ can lead to the formation of highly reactive hydroxyl radicals, which can damage cellular proteins, RNA, DNA, and lipids. Interaction of ROS with nitric oxide or fatty acids can lead also to the formation of peroxynitrite or peroxyl radicals, respectively, that are also highly reactive [38]. Although mitochondrial ROS (mROS) have been previously mainly considered as by-products of oxidative metabolism, it is now clearly established that they also act as important signaling molecules controlling a plethora of cellular functions, both in physiology and in pathology [40]. Mitochondrial Ca^2+^ uptake, by increasing the metabolic rate, and thus O_2_ consumption and respiratory chain electron leakage, drives superoxide production [38]. Ca^2+^ may promote mROS formation both directly, by stimulating mROS generating enzymes, like glycerol phosphate and α-ketoglutarate dehydrogenase [108], and indirectly, as in the case of nitric oxide synthase (NOS) activation that, by forming NO, blocks complex IV and leads to mROS formation [30]. In addition, the mild mitochondria uncoupling effect (Δ*Ψ*_m_ dissipation) of Ca^2+^ uptake contributes to mROS generation. Importantly, mROS play a crucial role in cancer progression, eliciting metabolic adaptations essential for metastasis formation and invasion [84]. It has been shown, in a triple-negative breast cancer model, that the inhibition of mitochondrial Ca^2+^ uptake causes a decrease in mROS production and consequently a reduction in cancer progression and metastasis formation [106].

Finally, mitochondrial Ca^2+^ overload triggers mitochondrial permeability transition pore (mPTP) opening (Fig. 1). mPTP is a high-conductance channel mediating mitochondrial swelling [4, 81]. Matrix Ca^2+^ is an essential permissive factor for mPTP opening: as [Ca^2+^]_cyt_ increases beyond a certain value, mitochondrial Ca^2+^ overload ensues. This, together with other causal factors, most notably oxidative stress, high phosphate concentrations, and low adenine nucleotide concentrations, triggers mitochondrial “permeability transition,” i.e., the mitochondrial membrane becomes permeable to any molecule less than 1.5 kDa in size. Consequent dissipation of the Δ*Ψ*_m_ leads to membrane depolarization, increased mROS generation, and decreased ATP production, eventually triggering apoptosis. The function of Ca^2+^ in apoptosis is particularly fascinating, since a small amount of cytochrome c released from mitochondria can bind to and promote Ca^2+^ conductance through IP_3_R. The increased cytosolic Ca^2+^ then triggers a massive exodus of cytochrome c from all mitochondria in the cell, thus activating caspases and nucleases that finalize the apoptotic process [10]. Recently, it has been proposed that mPTP forms from the F-ATP synthase through a strictly Ca^2+^-dependent mechanism [32–34]. However, the detailed mechanisms of mPTP activation are still debated and readers are referred to specific contributions on this topic [5, 12, 36, 37, 67].

## The molecular characterization of the MCU complex

**Fig. 2:**
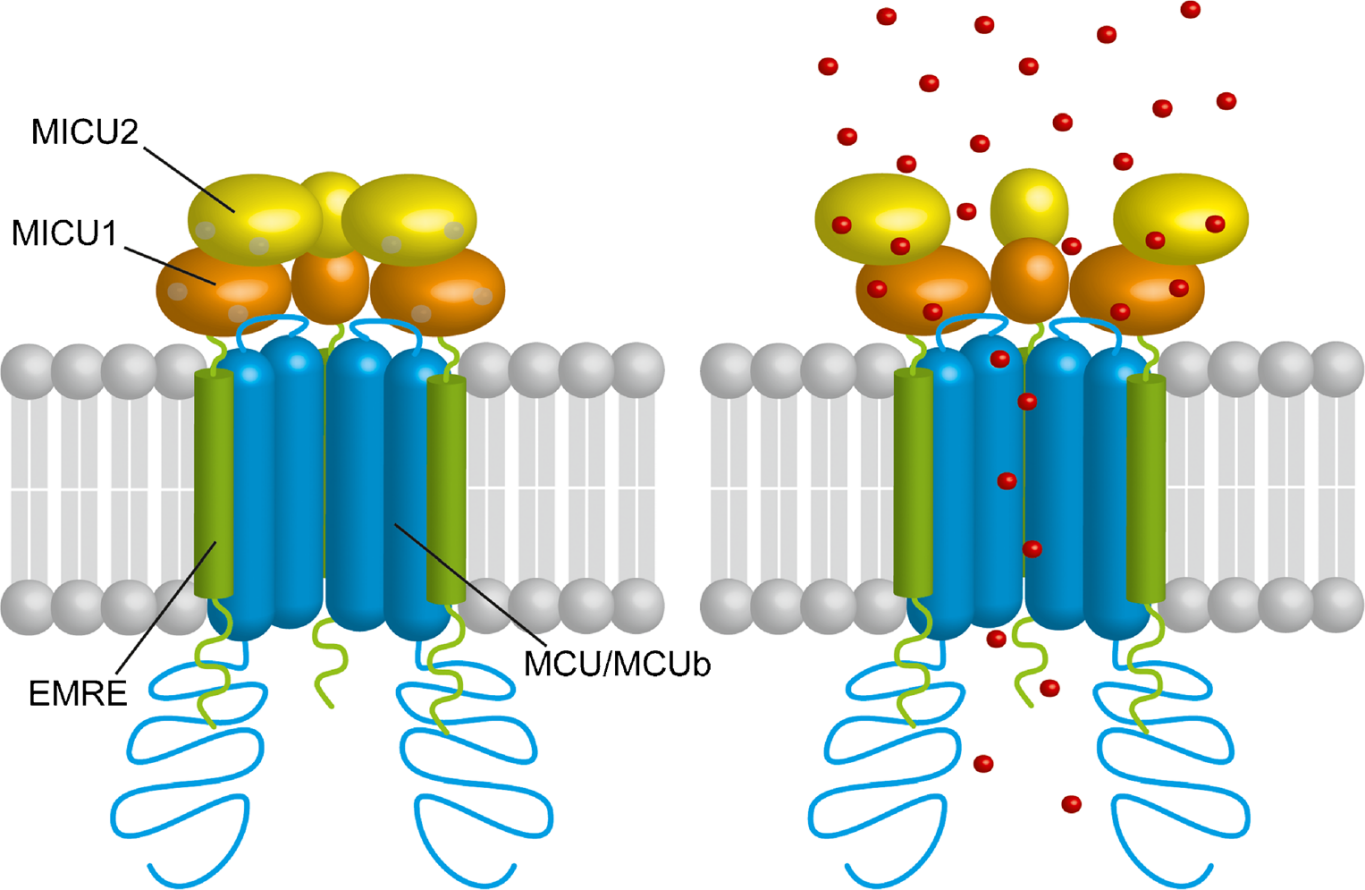
The mitochondrial Ca^2+^ uniporter is a complex composed of pore-forming proteins (comprising the channel subunit MCU, the dominant-negative subunit of the channel MCUb, and the short transmembrane regulator EMRE) and of regulatory proteins (MICU1 and MICU2). Both MICU1 and MICU2 contain EF-hand domains facing the intermembrane space. By sensing IMS [Ca^2+^], MICU1 and MICU2 coordinately regulate both the threshold and the cooperativity of channel opening

In 2011, the identification of the gene encoding the pore-forming subunit of the MCU, made by two independent groups [3, 21], marked a turning point in the field of mitochondrial physiology and paved the way for the characterization of one of the most sophisticated ion channels described so far. We will briefly describe the different components of the MCU complex distinguishing the membrane pore-forming subunits from the soluble regulatory components (Fig. 2).

### The pore-forming subunits

Three proteins have been identified as components of the MCU pore-forming subunit that spans the IMM: MCU, MCUb, and EMRE (Fig. 2).

The *MCU* gene, originally named *CCDC109a*, was identified through a bioinformatics screening of the MitoCarta database, i.e., a compendium of mitochondrial proteins identified by mass spectrometry analyses on mitochondrial preparations from different mouse tissues [3, 21, 72]. The *MCU* gene is well conserved among metazoan and plants while is absent in yeast, that lacks the Ruthenium red-sensitive mitochondrial Ca^2+^ uptake, in some fungi and in protozoans [8]. It encodes a 40 kDa protein that contains two predicted transmembrane domains joined by a very short, but highly conserved, loop that faces the intermembrane space (IMS). The N- and C-domains, which represent the majority of the protein sequence, face the mitochondrial matrix [3, 61]. The MCU protein structure analysis reveals two important aspects. First, since MCU displays only two transmembrane domains, it has to undergo oligomerization to form a functional channel. This is confirmed by blue native gel separation experiments of purified mitochondria, that display a high molecular complex containing MCU with an apparent molecular weight of 450 kDa [3, 21, 87]. Second, consensus sequences of classical Ca^2+^-binding domains have not been identified in MCU protein sequence. This suggests that MCU is unable to regulate its own activity. In addition, the MCU loop region that faces the IMS and that connects the two transmembrane domains is too small to contain regulatory elements since it is formed by a stretch of only 11 amino acids. Nonetheless, the loop includes salient residues necessary for MCU channeling. In particular, the loop contains the “DIME” motif, characterized by negatively charged amino acids (such as D260 and E263) essential to confer selectivity to the MCU channel [3, 21]. In addition, the residue S258 is critical to confer sensitivity of MCU complex to Ru360, the most potent inhibitor of the uniporter [3].

The protein structure of the N-terminal domain of MCU was resolved by a crystallographic study [51]. This domain contains a residue (S92) that was predicted as a putative phosphorylation site for CaMKII [51]. Mutation of this residue causes a reduction in the MCU Ca^2+^ conductance. This finding matches with the demonstration that mitoplasts derived from hearts treated with CaMKII inhibitors display a reduced MCU current [45].

Although MCU oligomer has been predicted to be a tetramer by a molecular dynamic approach [87], NMR and cryo-EM of the *Caenorhabditis elegans* MCU identified a pentamer complex [71]. In both of the proposed molecular structures, the DIME motifs form the pore entrance and they are part of the channel selectivity filter [71, 87]. Whether the expression and the purification of the *C. elegans* MCU in a prokaryotic system, which does not express the essential complex component EMRE (see below), are sufficient to ensure the correct structure assembling is unclear. Indeed, EMRE seems required for ensuring mitochondrial Ca^2+^ uptake and for the assembly of the regulatory subunits MICU1 and MICU2 [98], but its role on the folding of MCU, and thus for MCU structure, is still debated and needs further clarification.

The *MCUb* gene, formerly known as *CCDC109b*, was identified through an MCU sequence homology screening [87], and the incorporation of MCUb in the MCU complex has been demonstrated also by proteomics experiments [98]. MCUb greatly impairs Ca^2+^ permeation through the MCU [87]. It is present only in vertebrates, while it is absent in other organisms in which MCU is present. The MCUb amino acid sequence is highly conserved among different species and shares 50% of similarity with MCU [87]. For this reason, the overall predicted domain distribution and topology are conserved between MCU and MCUb. Nonetheless, MCUb presents salient differences from MCU. Firstly, two critical and conserved amino acid substitutions in the loop region confer to MCUb a dominant-negative function. Indeed, the substitution of the MCU loop residue E256 with a noncharged residue drastically reduces the conductivity of the channel [87]. In addition, MCU and MCUb show radically different expression profiles among tissues. Consequently, some tissues, such as the heart, exhibit a high MCU:MCUb ratio, while others, such as skeletal muscle, display a lower ratio. As for the physiological relevance of the presence and the differential expression of this isoform, it might represent one of the mechanisms that underlie the different MCU currents recorded in different tissues [29].

EMRE is the last component of the MCU pore to be identified. It is a 10 kDa protein of the IMM, and it represents a metazoan innovation since it is not present in the other eukaryotic taxa where MCU and MICU1 are expressed. It is composed of a transmembrane domain, a short N-terminal domain, and a highly conserved acidic C-terminal domain [98]. EMRE is essential for MCU activity, as demonstrated by experiments in EMRE KO cells where mitochondrial Ca^2+^ uptake is abolished. Even though in planar lipid bilayer MCU and the regulatory subunits MICU1 and MICU2 display the ability to interact with each other without the presence of EMRE [77], this protein has been proposed to play a fundamental role in the interaction between the pore core subunits and the regulatory subunits [98]. In addition, in yeast cells, that do not present mitochondrial Ca^2+^ uptake, human MCU is able to assemble in a functional channel only when EMRE is present. This gave rise to the concept that EMRE is essential to assemble a functional MCU channel in metazoan organisms [48]. Furthermore, the acidic C-terminal domain has been identified as a matrix-Ca^2+^ sensor that governs the MCU activity. Accordingly, EMRE would be able to form a unique regulatory complex with MICU1 and MICU2 that is able to sense Ca^2+^ at both sides of IMM [109]. This is in opposition to another report, which proposes a structural role of EMRE and a different topology across the IMM, incompatible with the suggested matrix-Ca^2+^ sensor of the acidic C-terminal domain [121].

### The regulatory subunits

As mentioned above, none Ca^2+^-sensing domains have been identified in the MCU structure, indicating that MCU is unable to regulate its own activity. Instead, it is clear that the regulation of MCU is dependent on IMS-residing proteins, namely MICU proteins. These belong to a family of proteins with common features: they are located in the mitochondria, they display EF-hand domains in their protein structure, and they interact with MCU [80] (Fig. 2). Through an integrative strategy that fuses comparative physiology, evolutionary genomics, organelle proteomics, and RNAi screenings, MICU1 was identified as a critical modulator of mitochondrial Ca^2+^ uptake even before the identification of MCU [80]. MICU1 is fundamental for the proper gatekeeping of the MCU channel, as demonstrated by the fact that the silencing of MICU1 causes mitochondrial Ca^2+^ overload [19, 59]. In addition, MICU1 acts also as cooperative activator of MCU, thus ensuring the increase in the MCU Ca^2+^ conductivity during cell stimulation [19].

Some years after MICU1 discovery, two MICU1 paralog genes were identified. These originate from a gene duplication event prior to vertebrate evolution. MICU2, formerly named EFHA1, displays a tissue expression pattern similar to MICU1 [83]. Instead, MICU3, formerly named EFHA2, is expressed prevalently in specific tissues, such as the nervous system and skeletal muscle [73, 83]. As MICU1, MICU2 displays two well-conserved EF-hand domains and several studies demonstrated that it is located in the IMS [19, 42]. Interestingly, MICU2 stability depends on the presence of MICU1, and the knockdown of MICU1 causes the destabilization of MICU2 protein, without affecting MICU2 mRNA levels [77, 83]. Indeed, MICU2 forms obligate heterodimers with MICU1, stabilized by a disulfide bond through two conserved cysteine residues [77], that have been hypothesized to be joined thanks to the mitochondrial oxidoreductase Mia40 [82]. The MICU1-MICU2 heterodimer is responsible for one of the most peculiar properties of mitochondrial Ca^2+^ uptake, i.e., the sigmoidal response to increasing [Ca^2+^]_cyt_ [19, 77]. In detail, in resting conditions, the MCU complex is inhibited to prevent mitochondrial Ca^2+^ overload and ion vicious cycles. However, when [Ca^2+^]_cyt_ increases, the MCU complex is subjected to a cooperative activation that ensures the prompt response of the mitochondria to cell challenge. Overall, the regulation of MCU complex activity by MICU1-MICU2 heterodimers is possible thanks to the ability of these proteins to sense Ca^2+^ concentration through their EF-hand domains [46, 77]. Nonetheless, the affinity for Ca^2+^ of the EF-hand domains is still controversial. As for MICU1, *K*_d_ measurements performed by isothermal titration calorimetry range from 4 to 40 μM [111, 115], while measurements of intrinsic tryptophan fluorescence record a higher affinity, with a *K*_d_ of ~ 300 nM [47]. These discrepancies, reflecting the different technical approaches, surely need further investigation. By in vivo experiments and electrophysiological studies carried out in planar lipid bilayer, our laboratory demonstrated that, at low [Ca^2+^]_cyt_, MICU2 inhibits MCU activity, thus representing the genuine gatekeeper of the channel. On the other hand, MICU1 senses high [Ca^2+^] and it allows the cooperative activation of MCU during cytosolic Ca^2+^ increases. Along these lines, MICU1 silencing causes Ca^2+^ overload due to the loss of the gatekeeper MICU2 and reduces the maximal activation of MCU due to the loss of cooperativity [27, 47, 77, 83, 111]. Nonetheless, the stoichiometry of MCU regulators in the complex is still completely unknown. This is a problematic issue, since it has been proposed that during Ca^2+^ stimulation MICU1 multimers undergo molecular rearrangement [113]. Furthermore, it has been recently proved that the ratio between MICU1 and MCU is sufficient to account for the different regulatory properties of the MCU complex in different tissues [73]. Indeed, the authors proposed that the different amounts of MCU not associated with MICU1 explain the tissue specificity of cytosolic Ca^2+^ transients decoding at the level of mitochondria. In particular, the low MICU1:MCU ratio measured in the heart allows mitochondrial Ca^2+^ uptake even for low [Ca^2+^]_cyt_ transients, due to a low gating of the channel accompanied by a low cooperativity. In this way, the beat-to-beat Ca^2+^ transients that occur in the heart cause an integrative Ca^2+^ accumulation. On the other hand, the high MICU1:MCU ratio, as observed in the liver, confers high cooperative activation of the channel but strengthens the threshold of activation. Therefore, subthreshold [Ca^2+^] fluctuations are not sufficient to trigger mitochondrial Ca^2+^ uptake. Moreover, each single sustained cytosolic Ca^2+^ increase is effectively transmitted to the mitochondria [73].

Other mitochondrial proteins have been identified as putative modulators of MCU activity. MCUR1, formerly named CCDC90a, was identified as a modulator of MCU, since the silencing of this protein causes a decrease of mitochondrial Ca^2+^ uptake in HEK293T cells [58]. However, its role in the MCU complex is highly debated, because it is also important for complex IV assembly and MCUR1 silencing causes a consistent drop of mitochondrial membrane potential [78]. The other component proposed to be part of the MCU complex is SLC25A23 [39], which belongs to a family of Mg-ATP/Pi solute carriers across IMM [2]. The mutation of its EF-hand domains reduces mitochondrial Ca^2+^ accumulation [39], but whether this depends on a direct MCU activity regulation or whether it affects mitochondrial bioenergetics or mitochondrial Ca^2+^ buffering capacity is still debated.

## Mitochondrial Ca^2+^ signaling in physiology: general framework and effects of MCU modulation

### Heart

Back in 1883, the heart was the first striated muscle that was demonstrated to contract in response to Ca^2+^ [90]. Much of the Ca^2+^ needed for contraction comes from the sarcoplasmic reticulum (SR) and is released in a beat-to-beat fashion by the process named Ca^2+^-induced Ca^2+^ release [7]. Indeed, it was postulated that, in mammalian cardiac muscle, the entry of a small amount of Ca^2+^ through the sarcolemma during the plateau of the action potential results in a large increase of intracellular Ca^2+^ through the RyR2, an event considered necessary for contraction (for a review, see [28]). Specifically, the depolarization induced by the action potential opens the L-type Ca^2+^ channels located on the membrane and transverse tubules, resulting in the entry of a small amount of Ca^2+^. This induces a large increase in the dyadic space, the region bounded by the t-tubule and SR [17]. This increase makes the SR RyR2s open, thereby releasing a much larger amount of Ca^2+^ from the SR. The latter event causes an increase in free Ca^2+^ ion concentration from approximately 100 nM to 1 μM, making more Ca^2+^ available for binding to troponin C (TnC). The binding of Ca^2+^ ions to TnC initiates a cascade of events leading to force generation by the cycling of cross-bridges, i.e., the interaction between the thin and thick filaments [17]. For relaxation to occur, Ca^2+^ must be removed from the cytoplasm. This requires the closure of RyR2s and, concomitantly, that Ca^2+^ is both pumped back into the SR, by the SERCA and out of the cell, largely by the Na^+^/Ca^2+^ exchanger (NCX), with some contribution from the plasma membrane Ca^2+^-ATPase [17]. Importantly, physiological sympathetic stimulation of the heart through β-adrenergic receptors increases the force of contraction (inotropy) and accelerates relaxation (lusitropy) [7]. The L-type Ca^2+^ channel is the main route for Ca^2+^ entry into cardiac myocytes that not only results in contraction but, importantly, in the upregulation of ATP production that powers cardiac excitation and contraction. Indeed, maintenance of intracellular Ca^2+^ homeostasis is critical for the regulation of mitochondrial ATP production [112]. Importantly, most of the ATP needed for cardiac excitation and contraction is synthesized within the mitochondria via oxidative phosphorylation that, as mentioned above, is a Ca^2+^-dependent process [23]. The fundamental role of the mitochondria in meeting changes in energy demand, such as upon increased workload or hormonal stimulation, is demonstrated by the close apposition of the mitochondria and the major source of Ca^2+^ for contraction, the SR. Therefore, it was hypothesized that Ca^2+^ release from the SR will elevate local Ca^2+^ to high levels resulting in a large mitochondrial Ca^2+^ influx [53]. Nevertheless, direct patch clamp recordings have shown that cardiac mitochondria I_MCU_ is substantially smaller than that of other tissues and, in particular, ~ 30 times smaller than skeletal muscle I_MCU_ [29]. In the heart, the mitochondria occupy 37% of cellular volume. Therefore, the small I_MCU_ might prevent excessive buffering of Ca^2+^ needed for contraction. Furthermore, excessive mitochondrial Ca^2+^ uptake, in conjunction with accumulation of ROS, has long been associated to the opening of the mPTP, leading to irreversible Δ*Ψ* collapse, swelling of the mitochondria, with consequent loss of cytochrome c and ultimately necrotic cardiomyocyte cell death, as observed in ischemic/reperfused myocardium [24]. Therefore, reducing the amplitude of cardiac mitochondrial transients might serve as a safety mechanism.

Despite the physical proximity of the mitochondria to the SR compartment and their Ca^2+^-dependent role in ATP production, the ability of the mitochondria to serve as significant dynamic buffers of cytosolic Ca^2+^ in the heart is still debated [13]. Furthermore, highly controversial is whether the fast cytosolic Ca^2+^ transients in excitation-contraction coupling in beating cardiomyocytes are transmitted to the matrix compartment in a beat-to-beat fashion or in a slow integration pattern [43]. This issue has been addressed by the first study reporting the effects of MCU modulation on heart function [26]. In detail, by means of a GFP-based Ca^2+^ indicator targeted to the OMM of neonatal cardiomyocytes, Pozzan and coworkers demonstrated the presence of microdomains of high [Ca^2+^] generated at the SR/mitochondria contacts that allow the massive entrance of Ca^2+^ into these organelles. Indeed, a fraction of Ca^2+^ released during systole enters the mitochondria and is released back into the cytoplasm during diastole, resulting in a significant buffering of Ca^2+^ peaks. In addition, the modulation of MCU protein levels by silencing or overexpression enhances or decreases the amplitude of cytoplasmic Ca^2+^ oscillation, respectively, and the opposite effect takes place into the mitochondria. Furthermore, mitochondrial Ca^2+^ uptake in the heart mitochondria is controlled by a low MICU1:MCU ratio, as discussed above. This property has been hypothesized to ensure beat-to-beat mitochondrial Ca^2+^ accumulation at low frequency, while allowing an integrative matrix Ca^2+^ accumulation when frequency increases [73].

Despite these findings demonstrating the importance of mitochondrial Ca^2+^ buffering in cardiac physiology, the heart phenotype of the first model of MCU knockout mouse was surprisingly mild [41, 75]. As expected, mitochondria isolated from MCU^−/−^ cardiomyocytes do not take up Ca^2+^ [75] and have lower resting Ca^2+^ levels compared to controls. However, basal ATP levels are unchanged, demonstrating a preserved basal mitochondrial energetics [41]. Furthermore, mice lacking MCU show normal basal cardiac function in terms of ejection fraction, fractional shortening, stroke volume, and chamber size, both in adulthood (12-month-old mice) and in aging (20-month-old mice). In addition, no differences between WT and MCU^−/−^ mice were observed in the left ventricular cardiac output at baseline and after isoproterenol stimulus, which mimics the “fight or flight” response, i.e., an episode of high-energy demand triggered by catecholamine-induced heart acceleration. Also, when mice were subjected to surgical transverse aortic constriction (TAC), as a model of chronic stress, MCU^−/−^ hearts showed the same cardiac parameters measured in the WT [41]. Taken together, these data suggest that mitochondrial Ca^2+^ accumulation is dispensable both for the basal cardiac function and during acute and chronic increased workload. Further experiments were carried out to assess the role of MCU during ischemia-reperfusion (I-R) injury. MCU^−/−^ hearts show no sign of I-R injury protection [75]. In detail, measurements of the rate pressure product and direct assessment of the infarct area in post-ischemic recovery period indicate no difference between MCU^−/−^ animals and the controls. Interestingly, when treated with cyclosporine A (CsA), that inhibits Ca^2+^-dependent cell death mediated by the opening of mPTP, WT hearts were protected from I-R injury, while MCU^−/−^ hearts were not. This result suggests that an mPTP-independent death pathway occurs in the absence of MCU [75]. It is noteworthy that the birth ratio of the MCU^−/−^ mice, which are in an outbred strain composed by a mix of CD1 and C57/BL6 backgrounds, is lower than expected [75] and that, in an inbred strain, MCU deletion is embryonically lethal [68]. These data demonstrate a crucial role of mitochondrial Ca^2+^ uptake during embryonic development, which is hidden in the mixed strain.

**Fig. 3:**
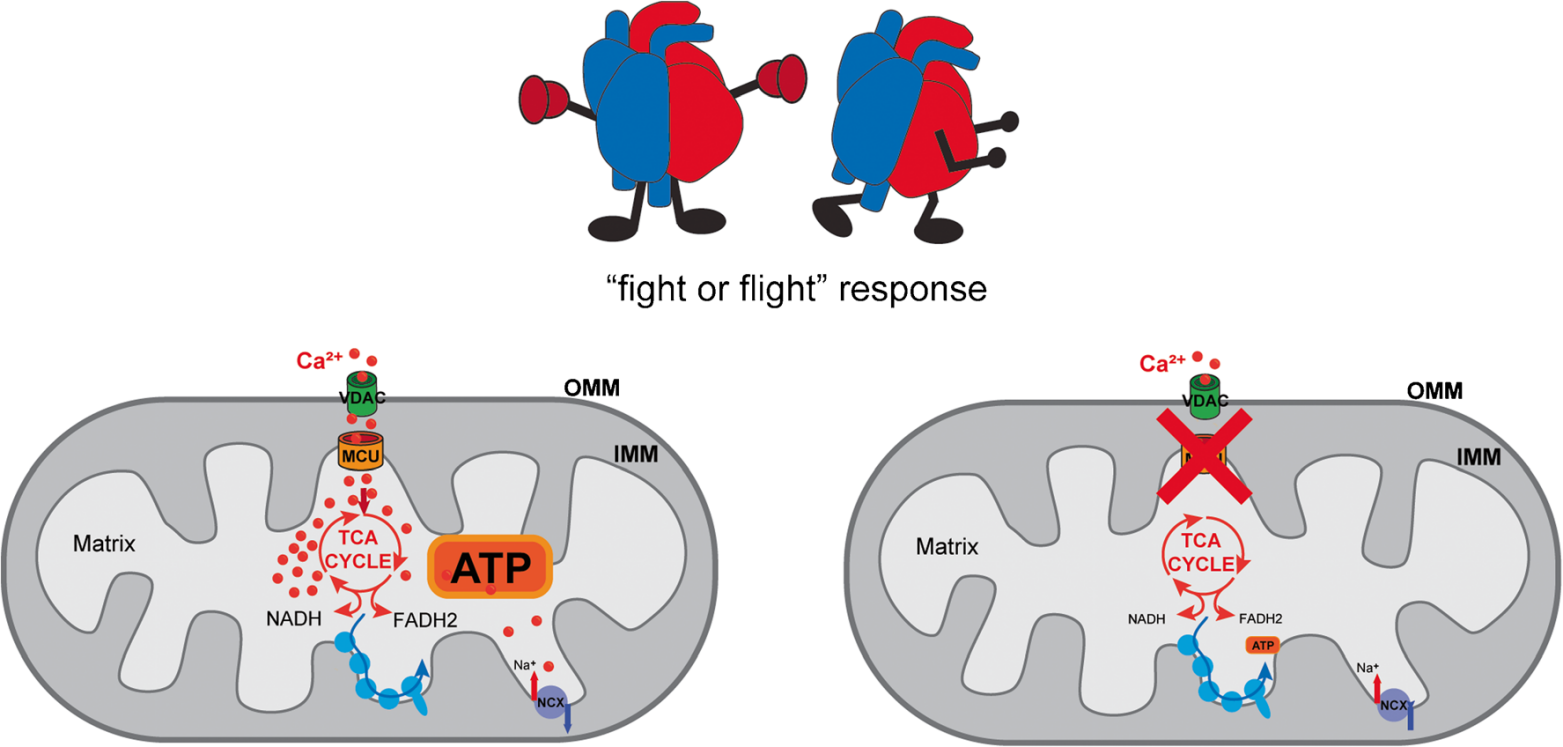
Dominant-negative MCU (DN-MCU) transgenic mice and inducible heart-specific MCU^−/−^ mice are characterized by impaired “fight or flight” response, due to lack of ATP production required for heart rate increase

The first heart-specific model generated was a transgenic mouse expressing a dominant-negative MCU isoform, MCU^D260Q,E263Q^ (DN-MCU), in the same mixed background of the constitutive MCU^−/−^ model [120]. When expressed in cultured cells, DN-MCU does not completely abolish organelle Ca^2+^ accumulation [21], although the mitochondria from DN-MCU-expressing hearts have no measurable mitochondrial Ca^2+^ uptake. DN-MCU mice have normal heart rate; however, they display an impairment in the “fight or flight” response (Fig. 3). In detail, when sinus atrial node (SAN) cells undergo an extreme physiological stress, ATP generation is required to fuel SERCA activity to maintain the proper Ca^2+^ load of the SR. DN-MCU mice are not able to increase the heart rate under physiological stress indicating that the ATP production is MCU dependent. ATP dialysis in cardiac pacemaker cells is sufficient to recover the phenotype. Additionally, oxygen consumption rate (OCR) is increased in DN-MCU-isolated perfused heart, but not in permeabilized fibers or isolated mitochondria [89]. Moreover, DN-MCU heart has a higher diastolic cytosolic [Ca^2+^], consistent with the loss of mitochondrial buffering. This cytosolic Ca^2+^ increase is partially rescued by the addition of ATP, suggesting that these cardiomyocytes display an extramitochondrial adaptation that depends on the reduced ATP availability. Importantly, similar to the MCU^−/−^ model, DN-MCU hearts are not protected against I-R injury.

**Fig. 4:**
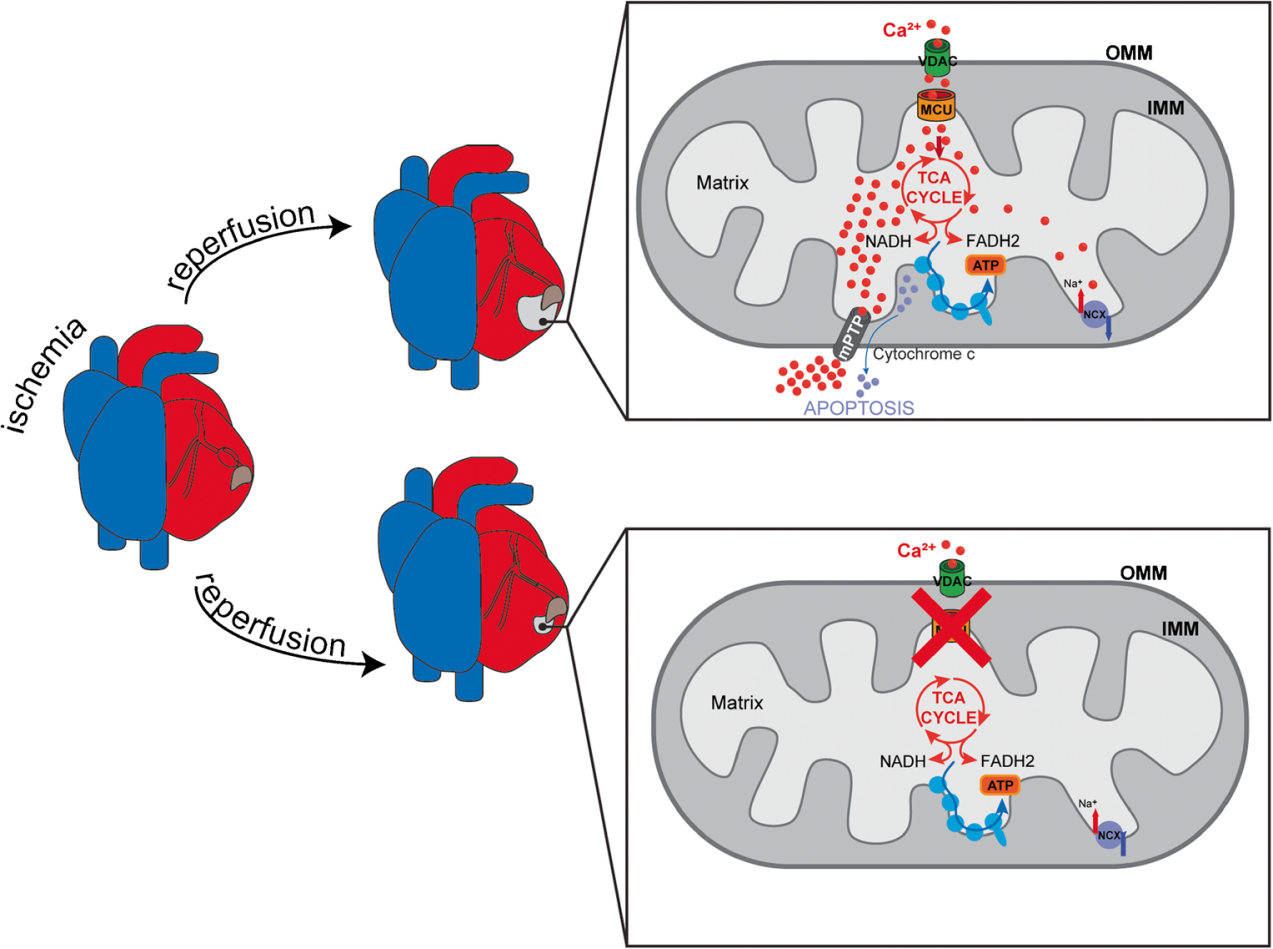
Inducible cardiac-specific MCU deletion confers protection from ischemia-reperfusion (I-R)-induced damage associated to mitochondrial Ca^2+^ overload and mPTP opening

Next, a mouse model with two LoxP sites flanking exons 5 and 6 of the MCU gene was mated with animals expressing a tamoxifen-inducible Cre recombinase driven by a cardiomyocyte-specific promoter. MCU gene deletion was induced in adult mice, and the cardiac function was evaluated [50, 54]. Firstly, MCU ablation in adult heart led to a great reduction in mitochondrial Ca^2+^ accumulation, although the basal mitochondrial [Ca^2+^] was not affected. Similar to DN-MCU mice and total MCU^−/−^ mice, cardiac-specific inducible MCU^−/−^ mice are indistinguishable from WT in normal conditions and after cardiac pressure overload. Cardiomyocytes derived from these mice present normal respiration rate in basal conditions, although a decrease in oxygen consumption rate was detected after isoproterenol treatment [50, 54]. However, as opposed to previously reported total MCU knockout and DN-MCU mouse models [75, 120], in the inducible cardiac-specific model, MCU ablation strongly protects hearts from I-R injury [50, 54] (Fig. 4). Finally, studies on the cardiac-specific adult MCU^−/−^ mouse confirmed the impairment in the “fight or flight” response triggered by β-adrenergic stimulation [50, 54] (Fig. 3), as observed in the DN-MCU transgenic mouse model [120].

Mitochondrial [Ca^2+^] is regulated by the coordinated activity of influx and efflux pathways [101]. To assess the contribution of mitochondrial Ca^2+^ efflux to heart pathophysiology, Elrod’s group recently developed an inducible mouse model with a cardiomyocyte-specific deletion of the Slc8b1 gene [55], which was previously demonstrated to encode the mitochondrial Na^+^/Ca^2+^ exchanger (NCLX) [74]. These mice present a severe phenotype. Indeed, the heart-specific deletion of the exchanger causes acute myocardial dysfunction and fulminant heart failure with a survival rate of only 13%. The hearts of the knockout mice display increased mass and cardiac fibrosis. In addition, echocardiographic analyses show ventricular dilatation and decreased left ventricular function. Finally, the NCLX^−/−^ hearts present a great sarcomere disorganization. Regarding the molecular mechanism, knockout adult cardiomyocytes show a faster rate of mitochondrial swelling compared to control, increased superoxide generation, and compromised sarcolemmal integrity. Heart-specific NCLX^−/−^ mice crossed with cyclophilin D (CypD)-null mice showed an almost complete rescue of the phenotype, demonstrating that the sudden death induced by Slc8b1 deletion was due to an mPTP-dependent mechanism. The mRNA expression of Slc8b1 and of MICU1 was increased in left ventricular biopsies of explanted failing hearts of transplant recipients [55]. To determine the biological relevance of these findings, a cardiac-restricted doxycycline-controlled NCLX overexpressing mouse model was generated (NCLX-Tg). NCLX-Tg adult cardiomyocytes present increased mitochondrial Ca^2+^ efflux compared to controls which is sufficient to reduce mPTP activity. In addition, NCLX-Tg hearts show a reduction in infarct size and enhanced contractile function upon I-R injury, suggesting a cardioprotective role of increased mitochondrial Ca^2+^ efflux [55]. These data demonstrate that mitochondrial Ca^2+^ efflux capacity is necessary for the maintenance of mitochondrial homeostasis and heart cell survival.

### Skeletal muscle

Ca^2+^ represents a powerful intracellular messenger in skeletal muscle fibers, being able not only to trigger contractions by binding to troponin C but also to activate protein phosphorylation or dephosphorylation by binding to calmodulin and stimulating substrate oxidation by the mitochondria [100].

The rise of Ca^2+^ in the sarcoplasm is a key requirement for skeletal muscle contraction. This event is initiated by the excitation-contraction coupling mechanism that couples muscular action potentials to myofibril contraction [99]. This process relies on a direct coupling between two proteins, the SR Ca^2+^-releasing channel ryanodine receptor (RyR1) and the voltage-gated L-type Ca^2+^ channels (dihydropyridine receptors, DHPRs), located on the sarcolemma of the transverse tubule. As the RyR1s open, Ca^2+^ is released from the SR and diffuses into the bulk cytoplasm generating a Ca^2+^ spark. The reversible binding of Ca^2+^ ions to TnC triggers the cross-bridge cycling, thus producing force. TnC, together with troponin T (TnT), troponin I (TnI), and tropomyosin (TM), forms a regulatory unit that controls the dependency from Ca^2+^ to muscle contraction [100].

Not only Ca^2+^ links excitation to contraction but also conjugates excitation to transcription, thus accounting for the huge molecular heterogeneity of muscle cells [100]. For example, binding of Ca^2+^ to calmodulin is known to activate signaling pathways typical of the slow-oxidative phenotype [100]. Indeed, Ca^2+^- and thus activity-dependent transcriptional regulation via calcineurin (calmodulin-dependent phosphatase 2A) and NFAT is associated with the translation of fast and slow motor neuron activity into muscle fiber type-specific transcriptional programs [16].

The cross-bridge cycle between myosin and actin is not only dependent on Ca^2+^ but also on ATP hydrolysis that liberates energy for the mechanical work. ATP consumption increases by approximately 100-fold during contraction, and thus, high demand cannot be fulfilled by the finite amount of ATP normally stored inside the muscle [35].

It has been extensively shown that the Ca^2+^ waves during contraction are transmitted to the mitochondria both in vitro [14] and in vivo [95], which respond by activating Ca^2+^-sensitive dehydrogenases that are key rate-controlling enzymes in the TCA cycle [23, 35]. This tight coupling is achieved by the mitochondria being located adjacent to Ca^2+^ stores (SR) and in proximity of release sites (Ca^2+^ release units [CRUs]) [11].

The role of mitochondrial Ca^2+^ uptake in skeletal muscle physiology is being vigorously investigated, as detailed hereafter. The MCU^−/−^ mice which, as explained above, are characterized by a mild phenotype, the most affected tissue is the skeletal muscle [75]. Specifically, resting matrix Ca^2+^ levels of skeletal muscle mitochondria of MCU^−/−^ mice are diminished by approximately 75% compared to controls. In addition, the phosphorylation levels of PDH are increased, and accordingly, PDH activity is decreased, in line with the Ca^2+^-dependent regulation of PDP1. In workload tests, MCU^−/−^ mice have significant impairment in exercise capacity, in line with the role of mitochondrial Ca^2+^ accumulation to regulate ATP production necessary to maintain a normal muscle functionality.

**Fig. 5:**
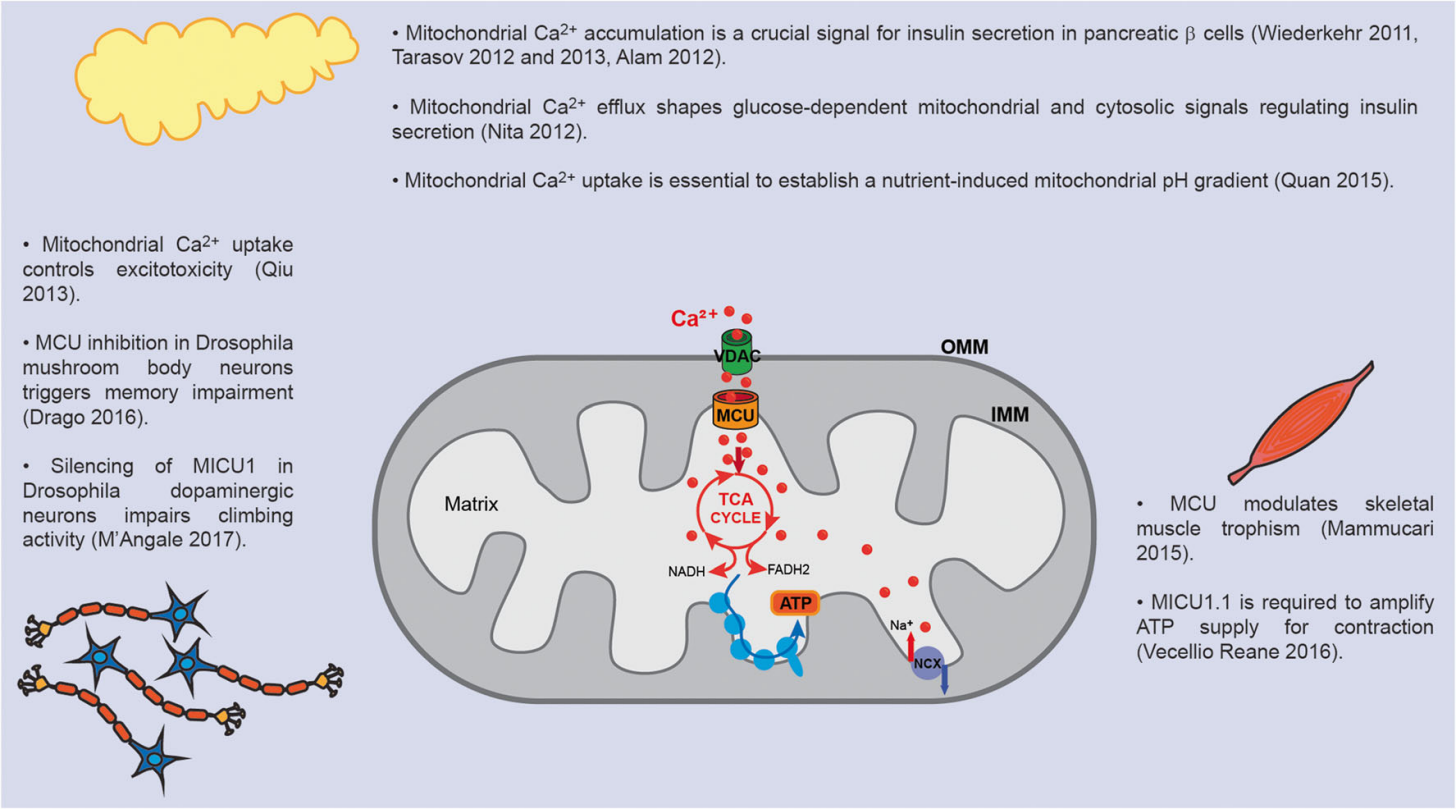
The importance of proper mitochondrial Ca^2+^ homeostasis in different organs, like the skeletal muscle, endocrine pancreas, and brain, is highlighted by the dysfunctional phenotype of specific animal models, as detailed in the figure

To avoid the compensatory effects acting during embryonic development, the role of mitochondrial Ca^2+^ homeostasis in skeletal muscle has been further investigated by local and postnatal administration of AAV (adeno-associated viral) vectors overexpressing or silencing MCU [60]. MCU overexpression and downregulation triggered muscle hypertrophy and atrophy, respectively (Fig. 5). Most importantly, MCU overexpression is protected from denervation-induced muscle atrophy caused by sciatic nerve excision. These effects are independent from the control of aerobic metabolism, as demonstrated by various lines of evidence. Firstly, PDH activity, although defective in MCU-silenced muscles, was unaffected in MCU overexpressing muscles. Second, hypertrophy was comparable in both oxidative and glycolytic muscles, and finally, semiquantitative analyses of aerobic metabolism revealed no major alterations. The control of skeletal muscle mass by mitochondrial Ca^2+^ modulation is due to the activity of two major hypertrophic pathways of skeletal muscle, PGC-1α4 and IGF1-AKT/PKB. Taken together, these results demonstrate that the modulation of mitochondrial Ca^2+^ accumulation after birth contributes to skeletal muscle trophism and that a Ca^2+^-dependent mitochondria-to-nucleus signaling route links organelle physiology to the control of muscle mass [60].

The control of oxidative phosphorylation by Ca^2+^ is particularly crucial in skeletal muscle, one of the most ATP-consuming organs of the body. It is thus not surprising that, compared to other tissues, skeletal muscle mitochondria display high Ca^2+^ conductance [29] and that skeletal muscle expresses a unique MCU Ca^2+^ uptake machinery [111]. Indeed, recently, an alternative splice variant of MICU1, that was named MICU1.1, was discovered [111]. This isoform, characterized by the addition of a micro-exon coding for four amino acids, greatly modifies the properties of the MCU. In detail, MICU1.1 binds Ca^2+^ one order of magnitude more efficiently than MICU1 and, when heterodimerized with MICU2, activates MCU current at lower Ca^2+^ concentrations than MICU1-MICU2 heterodimers. In vivo injection of antisense oligonucleotides mediating exon skipping of the MICU1.1 extra exon, and thus forced expression of MICU1, demonstrated that MICU1.1 is required for maintaining sufficient levels of mitochondrial Ca^2+^ uptake to provide the ATP needed for contraction [111] (Fig. 5). These results demonstrate a novel mechanism of the molecular plasticity of the MCU Ca^2+^ uptake machinery. Future studies will likely unravel other tissue-specific regulatory mechanisms of mitochondrial Ca^2+^ uptake.

### Pancreatic β cells

The initial concept that Ca^2+^ controls the release of insulin by β cells goes back to the seminal observation that the release of this hormone is blocked in the absence of Ca^2+^ [119]. Notably, β cells, the sole source of circulating insulin, convert small fluctuations in blood glucose concentration into large changes in insulin secretion within minutes. These cells are electrically excitable cells that respond to increases in glucose concentration with enhanced metabolism, closure of ATP-sensitive K^+^ channels, and electrical spiking [97]. In detail, glucose induces the secretion of insulin through the stimulation of oxidative metabolism, an elevation in cytosolic ATP/ADP ratio, and the closure of ATP-sensitive K^+^ channels (KATP). The subsequent depolarization of the plasma membrane results in oscillatory Ca^2+^ influx through voltage-gated Ca^2+^ channels, which is the main and necessary signal for insulin release through secretory granule exocytosis [31, 118]. Importantly, defects in the generation of Ca^2+^ oscillations, and thus in pulsatile insulin secretion, are associated with the loss of normal glucose homeostasis in type 2 diabetes [103]. Ca^2+^ influx through voltage-dependent Ca^2+^ channels has been shown to create Ca^2+^ microdomains beneath the β-cell plasma membrane with high [Ca^2+^] that might be crucial for insulin exocytosis and to open Ca^2+^-activated K^+^ channels (see below and [96]). Therefore, in pancreatic β cells, ATP acts as a signaling molecule initiating plasma membrane electrical activity linked to Ca^2+^ influx. Of note, the mitochondria play a central role in this process by connecting glucose metabolism to insulin release [57]. Specifically, in single primary β cells, cytosolic Ca^2+^ oscillations triggered by electrical stimulation cause stable increases in both [Ca^2+^]_m_ and cytosolic ATP/ADP ratio which depend on MCU activity [105]. Respiratory chain inhibitors and uncouplers strongly inhibit insulin release [44], and chelation of mitochondrial matrix Ca^2+^ [117] or silencing of either MICU1 or MCU [1, 104] causes defective insulin secretion in β-cell lines (Fig. 5). Finally, it has been proposed that mitochondrial Ca^2+^ accumulation is essential to establish a nutrient-induced mitochondrial pH gradient which is crucial to sustain ATP synthesis and metabolism secretion coupling in insulin-releasing cells [86]. In summary, a two-phase model has been proposed, according to which soon after glucose stimulation, cytosolic [ATP] increases independently of any increase in cytosolic or mitochondrial [Ca^2+^]. Subsequently, an increase in [Ca^2+^]_cyt_ occurs, which is followed by a rise in mitochondrial Ca^2+^ signal. This, in turn, stimulates mitochondrial metabolism and therefore ATP production. Importantly, mitochondrial Ca^2+^ efflux mediated by the Na^+^/Ca^2+^ exchanger NCLX contributes to the regulation of insulin secretion in β cells by shaping glucose-dependent mitochondrial and cytosolic Ca^2+^ signals [70] (Fig. 5).

### Neurons

Neurons require extremely precise spatial-temporal control of Ca^2+^-dependent processes since they regulate vital functions such as transmission of depolarizing signals, synaptic plasticity, and metabolism [15]. Neurons have thus developed complex pathways to couple Ca^2+^ signals to their physiological response. Therefore, neurons are extremely sensitive to [Ca^2+^] levels and even small defects in Ca^2+^ homeostasis can lead to destructive consequences and alter normal neuronal activity, such as in aging [49] and neurodegeneration [15]. Ca^2+^ influx into neurons occurs through plasma membrane receptors and voltage-dependent ion channels. Furthermore, the release of Ca^2+^ from the ER also contributes to the elevation of [Ca^2+^]_cyt_. Overall, a complex and highly compartmentalized Ca^2+^ signaling system in neuronal cells allows the activation of different spatially separated Ca^2+^-dependent processes at the same time [79]. The wide range of neuronal functions regulated by intracellular Ca^2+^ signals raises the question of how selectivity is encoded by such a universal messenger. One answer to this question is the presence of local Ca^2+^ signals, or Ca^2+^ microdomains, developed rapidly near open Ca^2+^ channels, creating spatial Ca^2+^ gradients of high [Ca^2+^] near the open pores [69]. The major sources of intracellular Ca^2+^ include Ca^2+^ influx through ligand-gated glutamate receptors, such as *N*-methyl-d-aspartate (NMDA) receptor (NMDAR) or various voltage-dependent Ca^2+^ channels (VDCCs), as well as the release of Ca^2+^ from intracellular stores [6]. The relative contribution of these sources will depend on neuron size, transmitter system, and location in neural circuits (i.e., excitatory or inhibitory) [6].

As for the Ca^2+^ entry through neuron plasma membrane channels, the activation of presynaptic neurons leads to the release of neurotransmitters into the synaptic cleft via the entry of Ca^2+^ through the voltage-operated Ca^2+^ channels (VOCCs). The released neurotransmitters in the synaptic cleft, in turn, activate receptors in the postsynaptic PM, thus initiating signal transmission. In postsynaptic neurons, the activation of neurotransmitter receptors results in the generation of Ca^2+^ signals that trigger responses that are specific to the type of receptor (reviewed in [15]).

Importantly, in addition to regulating the physiological functions of mature neurons, Ca^2+^ signaling also plays essential roles in the neurogenesis from neural stem/progenitor cells which proliferate, migrate, and ultimately differentiate into billions of neurons and glia that populate the brain. There is increasing evidence that Ca^2+^ signaling controls specific genetic programs that establish the structures of the nervous system through Ca^2+^-dependent signaling pathways such as the calcineurin-NFAT signaling axis that has been shown to be critical for axonal growth as well as presynaptic development, dendritogenesis, and neuronal survival (reviewed in [107]).

Neurons are responsible for a huge oxygen consumption in resting conditions in humans. Indeed, the brain uses about 20% of the total oxygen consumed at rest but represents only 2% of the body mass [64]. Importantly, neurons are almost exclusively dependent on mitochondrial oxidative phosphorylation (OXPHOS) as a main source of ATP, and Ca^2+^ entry into the mitochondria guarantees activity-dependent regulation of cellular energy metabolism [52]. As for skeletal muscle contraction [35], also neuronal activity not only contributes significantly to ATP consumption but also rapidly adapts to increased activity stimulating ATP synthesis through a Ca^2+^-dependent increase in OXPHOS [88].

The mitochondria exert other neuron-specific functions. Indeed, their cellular distribution contributes to the accumulation of a large amount of Ca^2+^ in a defined subcellular domain, promoting large local cytoplasmic Ca^2+^ rises. Importantly, Ca^2+^ sequestration by the mitochondria profoundly affects neurotransmitter release, being strategically located in the proximity of Ca^2+^ channels such as NMDAR at the synaptic terminal [9, 62]. In general, mitochondria recruitment to neuronal soma, synapses, and dendritic spines is crucial for the regulation of nerve activity, and any change in the positioning of the mitochondria to subcellular domains affects neuron physiology and might contribute to the pathogenesis of neurodegeneration [102].

For a long time, one of the outstanding questions among neurologists has been whether modulation of mitochondrial Ca^2+^ uptake impinges on the neurotoxic effects of the excitatory neurotransmitter glutamate. In this respect, it was clear that the cause of neuronal dysfunction and death was the excessive Ca^2+^ influx in neuron through the NMDA subtype of glutamate receptor. Experiments performed by modulating MCU allowed to directly determine the role of mitochondrial Ca^2+^ uptake in response to excitotoxic stimuli [85]. In detail, MCU overexpression exacerbated NMDA-induced loss of mitochondrial membrane potential and cell death, while MCU knockdown protected the mitochondria from NMDA-induced depolarization and increased resistance to excitotoxicity. Endogenous MCU expression is controlled by neuroprotective synaptic activity, which negatively regulates MCU transcription, with a mechanism that implies nuclear Ca^2+^ and CaM kinase-dependent induction of the transcription factor Npas4 [85] (Fig. 5).

Following a report of a siRNA library screening that identified MCU and MICU1 as important factors for proper memory formation [114], a study performed in *Drosophila melanogaster* established that MCU-mediated mitochondrial Ca^2+^ uptake during development is of fundamental importance for olfactory memory formation but not for learning. Decreased mitochondrial Ca^2+^ accumulation, triggered by overexpression of a dominant-negative isoform of MCU in mushroom body neurons, causes axon lengthening accompanied by decreased synaptic vesicle content [25]. A recent report focused on the role of mitochondrial Ca^2+^ uptake in dopaminergic neurons. Silencing of *D. melanogaster* CG4495 gene, identified as MICU1 homolog, in dopaminergic neurons impaired climbing activity, which was worsened with aging, and shortened life span [56] (Fig. 5).

## Concluding remarks

The physiological role of mitochondrial Ca^2+^ uptake has been extensively studied in the last few years thanks to the availability of transgenic animal models. Either constitutive or conditional deletion of MCU, as well as overexpression of MCUb, has been achieved in vivo. In addition, tissue-specific transgenic animals have been produced. The effects of MCU activity modulation on organs particularly relying on mitochondrial metabolism for their energy demand, including the heart, skeletal muscle, neurons, and pancreas, have been dissected. These studies have highlighted the importance of MCU in physiologic organ functions and in the protection from damaging insults, but also the existence of compensatory mechanisms. Intriguingly, cardiac-specific deletion or overexpression of the NCLX demonstrates the essential role of proper mitochondrial Ca^2+^ efflux for heart function and, in general, the requirement of fine-tuned mitochondrial Ca^2+^ dynamics for proper organ physiology. In the future, comprehensive studies will unravel still obscure aspects of mitochondrial Ca^2+^ homeostasis on cell and tissue functions.

## References

[1] Alam MR, Groschner LN, Parichatikanond W, Kuo L, Bondarenko AI, Rost R, Waldeck-Weiermair M, Malli R, Graier WF (2012). Mitochondrial Ca2+ uptake 1 (MICU1) and mitochondrial Ca2+ uniporter (MCU) contribute to metabolism-secretion coupling in clonal pancreatic β-cells. J Biol Chem.

[2] Bassi MT, Manzoni M, Bresciani R, Pizzo MT, Della Monica A, Barlati S, Monti E, Borsani G (2005). Cellular expression and alternative splicing of SLC25A23, a member of the mitochondrial Ca2+-dependent solute carrier gene family. Gene.

[3] Baughman JM, Perocchi F, Girgis HS, Plovanich M, Belcher-Timme CA, Sancak Y, Bao XR, Strittmatter L, Goldberger O, Bogorad RL, Koteliansky V, Mootha VK (2011). Integrative genomics identifies MCU as an essential component of the mitochondrial calcium uniporter. Nature.

[4] Bernardi P, Vassanelli S, Veronese P, Colonna R, Szabó I, Zoratti M (1992). Modulation of the mitochondrial permeability transition pore. Effect of protons and divalent cations. J Biol Chem.

[5] Bernardi P, Rasola A, Forte M, Lippe G (2015). The mitochondrial permeability transition pore: channel formation by F-ATP synthase, integration in signal transduction, and role in pathophysiology. Physiol Rev.

[6] Berridge MJ (1998). Neuronal calcium signaling. Neuron.

[7] Bers DM (2002). Cardiac excitation–contraction coupling. Nature.

[8] Bick AG, Calvo SE, Mootha VK (2012). Evolutionary diversity of the mitochondrial calcium uniporter. Science.

[9] Billups B, Forsythe ID (2002). Presynaptic mitochondrial calcium sequestration influences transmission at mammalian central synapses. J Neurosci.

[10] Boehning D, Patterson RL, Sedaghat L, Glebova NO, Kurosaki T, Snyder SH (2003). Cytochrome c binds to inositol (1,4,5) trisphosphate receptors, amplifying calcium-dependent apoptosis. Nat Cell Biol.

[11] Boncompagni S, Rossi AE, Micaroni M, Beznoussenko GV, Polishchuk RS, Dirksen RT, Protasi F (2009). Mitochondria are linked to calcium stores in striated muscle by developmentally regulated tethering structures. Mol Biol Cell.

[12] Bonora M, Morganti C, Morciano G, Pedriali G, Lebiedzinska-Arciszewska M, Aquila G, Giorgi C, Rizzo P, Campo G, Ferrari R, Kroemer G, Wieckowski MR, Galluzzi L, Pinton P (2017). Mitochondrial permeability transition involves dissociation of F _1_ F _O_ ATP synthase dimers and C-ring conformation. EMBO Rep.

[13] Boyman L, Chikando AC, Williams GSB, Khairallah RJ, Kettlewell S, Ward CW, Smith GL, Kao JPY, Lederer WJ (2014). Calcium movement in cardiac mitochondria. Biophys J.

[14] Brini M, De Giorgi F, Murgia M, Marsault R, Massimino ML, Cantini M, Rizzuto R, Pozzan T (1997). Subcellular analysis of Ca2+ homeostasis in primary cultures of skeletal muscle myotubes. Mol Biol Cell.

[15] Brini M, Calì T, Ottolini D, Carafoli E (2014). Neuronal calcium signaling: function and dysfunction. Cell Mol Life Sci.

[16] Calabria E, Ciciliot S, Moretti I, Garcia M, Picard A, Dyar KA, Pallafacchina G, Tothova J, Schiaffino S, Murgia M (2009). NFAT isoforms control activity-dependent muscle fiber type specification. Proc Natl Acad Sci U S A.

[17] Chung J-H, Biesiadecki BJ, Ziolo MT, Davis JP, Janssen PML (2016). Myofilament calcium sensitivity: role in regulation of in vivo cardiac contraction and relaxation. Front Physiol.

[18] Colombini M (2016). The VDAC channel: molecular basis for selectivity. Biochim Biophys Acta - Mol Cell Res.

[19] Csordás G, Golenár T, Seifert EL, Kamer KJ, Sancak Y, Perocchi F, Moffat C, Weaver D, de la Fuente Perez S, Bogorad R, Koteliansky V, Adijanto J, Mootha VK, Hajnóczky G (2013). MICU1 controls both the threshold and cooperative activation of the mitochondrial Ca2+ uniporter. Cell Metab.

[20] Das AM, Harris DA (1990). Control of mitochondrial ATP synthase in heart cells: inactive to active transitions caused by beating or positive inotropic agents. Cardiovasc Res.

[21] De Stefani D, Raffaello A, Teardo E, Szabò I, Rizzuto R (2011). A forty-kilodalton protein of the inner membrane is the mitochondrial calcium uniporter. Nature.

[22] Deluca HF, Engstrom GW (1961). Calcium uptake by rat kidney mitochondria. Proc Natl Acad Sci U S A.

[23] Denton RM (2009). Regulation of mitochondrial dehydrogenases by calcium ions. Biochim Biophys Acta.

[24] Di Lisa F, Bernardi P (2009). A CaPful of mechanisms regulating the mitochondrial permeability transition. J Mol Cell Cardiol.

[25] Drago I, Davis RL (2016). Inhibiting the mitochondrial calcium uniporter during development impairs memory in adult Drosophila. Cell Rep.

[26] Drago I, De Stefani D, Rizzuto R, Pozzan T (2012). Mitochondrial Ca2+ uptake contributes to buffering cytoplasmic Ca2+ peaks in cardiomyocytes. Proc Natl Acad Sci.

[27] Eisner V, Csordas G, Hajnoczky G (2013). Interactions between sarco-endoplasmic reticulum and mitochondria in cardiac and skeletal muscle—pivotal roles in Ca2+ and reactive oxygen species signaling. J Cell Sci.

[28] Eisner DA, Caldwell JL, Kistamás K, Trafford AW (2017). Calcium and excitation-contraction coupling in the heart. Circ Res.

[29] Fieni F, Lee SB, Jan YN, Kirichok Y (2012). Activity of the mitochondrial calcium uniporter varies greatly between tissues. Nat Commun.

[30] Ghafourifar P, Schenk U, Klein SD, Richter C (1999). Mitochondrial nitric-oxide synthase stimulation causes cytochrome c release from isolated mitochondria. Evidence for intramitochondrial peroxynitrite formation. J Biol Chem.

[31] Gilon P, Chae H-Y, Rutter GA, Ravier MA (2014). Calcium signaling in pancreatic β-cells in health and in type 2 diabetes. Cell Calcium.

[32] Giorgio V, von Stockum S, Antoniel M, Fabbro A, Fogolari F, Forte M, Glick GD, Petronilli V, Zoratti M, Szabo I, Lippe G, Bernardi P (2013). Dimers of mitochondrial ATP synthase form the permeability transition pore. Proc Natl Acad Sci.

[33] Giorgio V, Burchell V, Schiavone M, Bassot C, Minervini G, Petronilli V, Argenton F, Forte M, Tosatto S, Lippe G, Bernardi P (2017). Ca ^2+^ binding to F-ATP synthase β subunit triggers the mitochondrial permeability transition. EMBO Rep.

[34] Giorgio V, Guo L, Bassot C, Petronilli V, Bernardi P (2017) Calcium and regulation of the mitochondrial permeability transition. Cell Calcium. 10.1016/j.ceca.2017.05.004 10.1016/j.ceca.2017.05.004 28522037

[35] Glancy B, Willis WT, Chess DJ, Balaban RS (2013). Effect of calcium on the oxidative phosphorylation cascade in skeletal muscle mitochondria. Biochemistry.

[36] He J, Carroll J, Ding S, Fearnley IM, Walker JE (2017). Permeability transition in human mitochondria persists in the absence of peripheral stalk subunits of ATP synthase. Proc Natl Acad Sci.

[37] He J, Ford HC, Carroll J, Ding S, Fearnley IM, Walker JE (2017). Persistence of the mitochondrial permeability transition in the absence of subunit c of human ATP synthase. Proc Natl Acad Sci.

[38] Hempel N, Trebak M (2017). Crosstalk between calcium and reactive oxygen species signaling in cancer. Cell Calcium.

[39] Hoffman NE, Chandramoorthy HC, Shanmughapriya S, Zhang XQ, Vallem S, Doonan PJ, Malliankaraman K, Guo S, Rajan S, Elrod JW, Koch WJ, Cheung JY, Madesh M (2014). SLC25A23 augments mitochondrial Ca2+ uptake, interacts with MCU, and induces oxidative stress-mediated cell death. Mol Biol Cell.

[40] Holmström KM, Finkel T (2014). Cellular mechanisms and physiological consequences of redox-dependent signalling. Nat Rev Mol Cell Biol.

[41] Holmström KM, Pan X, Liu JC, Menazza S, Liu J, Nguyen TT, Pan H, Parks RJ, Anderson S, Noguchi A, Springer D, Murphy E, Finkel T (2015). Assessment of cardiac function in mice lacking the mitochondrial calcium uniporter. J Mol Cell Cardiol.

[42] Hung V, Zou P, Rhee H-W, Udeshi ND, Cracan V, Svinkina T, Carr SA, Mootha VK, Ting AY (2014). Proteomic mapping of the human mitochondrial intermembrane space in live cells via ratiometric APEX tagging. Mol Cell.

[43] Hüser J, Blatter LA, Sheu S-S (2000). Mitochondrial calcium in heart cells: beat-to-beat oscillations or slow integration of cytosolic transients?. J Bioenerg Biomembr.

[44] Hutton JC, Sener A, Herchuelz A, Atwater I, Kawazu S, Boschero AC, Somers G, Devis G, Malaisse WJ (1980). Similarities in the stimulus-secretion coupling mechanisms of glucose- and 2-keto acid-induced insulin release*. Endocrinology.

[45] Joiner MA, Koval OM, Li J, He BJ, Allamargot C, Gao Z, Luczak ED, Hall DD, Fink BD, Chen B, Yang J, Moore SA, Scholz TD, Strack S, Mohler PJ, Sivitz WI, Song L-S, Anderson ME (2012). CaMKII determines mitochondrial stress responses in heart. Nature.

[46] Kamer KJ, Mootha VK (2014). MICU1 and MICU2 play nonredundant roles in the regulation of the mitochondrial calcium uniporter. EMBO Rep.

[47] Kamer KJ, Grabarek Z, Mootha VK (2017). High-affinity cooperative Ca2+ binding by MICU1–MICU2 serves as an on–off switch for the uniporter. EMBO Rep.

[48] Kovács-Bogdán E, Sancak Y, Kamer KJ, Plovanich M, Jambhekar A, Huber RJ, Myre MA, Blower MD, Mootha VK (2014). Reconstitution of the mitochondrial calcium uniporter in yeast. Proc Natl Acad Sci U S A.

[49] Kumar A, Bodhinathan K, Foster TC (2009). Susceptibility to calcium dysregulation during brain aging. Front Aging Neurosci.

[50] Kwong JQ, Lu X, Correll RN, Schwanekamp JA, Vagnozzi RJ, Sargent MA, York AJ, Zhang J, Bers DM, Molkentin JD (2015). The mitochondrial calcium uniporter selectively matches metabolic output to acute contractile stress in the heart. Cell Rep.

[51] Lee Y, Min CK, Kim TG, Song HK, Lim Y, Kim D, Shin K, Kang M, Kang JY, Youn H-S, Lee J-G, An JY, Park KR, Lim JJ, Kim JH, Kim JH, Park ZY, Kim Y-S, Wang J, Kim DH, Eom SH (2015). Structure and function of the N-terminal domain of the human mitochondrial calcium uniporter. EMBO Rep.

[52] Llorente-Folch I, Rueda CB, Pardo B, Szabadkai G, Duchen MR, Satrustegui J (2015). The regulation of neuronal mitochondrial metabolism by calcium. J Physiol.

[53] Lu X, Ginsburg KS, Kettlewell S, Bossuyt J, Smith GL, Bers DM (2013). Measuring local gradients of intramitochondrial [Ca(2+)] in cardiac myocytes during sarcoplasmic reticulum Ca(2+) release. Circ Res.

[54] Luongo TS, Lambert JP, Yuan A, Zhang X, Gross P, Song J, Shanmughapriya S, Gao E, Jain M, Houser SR, Koch WJ, Cheung JY, Madesh M, Elrod JW (2015). The mitochondrial calcium uniporter matches energetic supply with cardiac workload during stress and modulates permeability transition. Cell Rep.

[55] Luongo TS, Lambert JP, Gross P, Nwokedi M, Lombardi AA, Shanmughapriya S, Carpenter AC, Kolmetzky D, Gao E, van Berlo JH, Tsai EJ, Molkentin JD, Chen X, Madesh M, Houser SR, Elrod JW (2017). The mitochondrial Na(+)/Ca(2+) exchanger is essential for Ca(2+) homeostasis and viability. Nature.

[56] M’Angale PG, Staveley BE (2017) Inhibition of mitochondrial calcium uptake 1 in Drosophila neurons. Genet Mol Res 16. 10.4238/gmr16019436 10.4238/gmr16019436 28198506

[57] Maechler P, Wollheim CB (2001). Mitochondrial function in normal and diabetic β-cells. Nature.

[58] Mallilankaraman K, Cárdenas C, Doonan PJ, Chandramoorthy HC, Irrinki KM, Golenár T, Csordás G, Madireddi P, Yang J, Müller M, Miller R, Kolesar JE, Molgó J, Kaufman B, Hajnóczky G, Foskett JK, Madesh M (2012). MCUR1 is an essential component of mitochondrial Ca2+ uptake that regulates cellular metabolism. Nat Cell Biol.

[59] Mallilankaraman K, Doonan P, Cárdenas C, Chandramoorthy HC, Müller M, Miller R, Hoffman NE, Gandhirajan RK, Molgó J, Birnbaum MJ, Rothberg BS, Mak D-OD, Foskett JK, Madesh M (2012). MICU1 is an essential gatekeeper for MCU-mediated mitochondrial Ca2+ uptake that regulates cell survival. Cell.

[60] Mammucari C, Gherardi G, Zamparo I, Raffaello A, Boncompagni S, Chemello F, Cagnin S, Braga A, Zanin S, Pallafacchina G, Zentilin L, Sandri M, De Stefani D, Protasi F, Lanfranchi G, Rizzuto R (2015). The mitochondrial calcium uniporter controls skeletal muscle trophism in vivo. Cell Rep.

[61] Martell JD, Deerinck TJ, Sancak Y, Poulos TL, Mootha VK, Sosinsky GE, Ellisman MH, Ting AY (2012). Engineered ascorbate peroxidase as a genetically encoded reporter for electron microscopy. Nat Biotechnol.

[62] Medler K, Gleason EL (2002). Mitochondrial Ca(2+) buffering regulates synaptic transmission between retinal amacrine cells. J Neurophysiol.

[63] Mildaziene V, Baniene R, Nauciene Z, Bakker BM, Brown GC, Westerhoff HV, Kholodenko BN (1995). Calcium indirectly increases the control exerted by the adenine nucleotide translocator over 2-oxoglutarate oxidation in rat heart mitochondria. Arch Biochem Biophys.

[64] Mink JW, Blumenschine RJ, Adams DB (1981). Ratio of central nervous system to body metabolism in vertebrates: its constancy and functional basis. Am J Phys.

[65] Mitchell P (1961). Coupling of phosphorylation to electron and hydrogen transfer by a chemi-osmotic type of mechanism. Nature.

[66] Mitchell P (1966). Chemiosmotic coupling in oxidative and photosynthetic phosphorylation. Biol Rev Camb Philos Soc.

[67] Morciano G, Giorgi C, Bonora M, Punzetti S, Pavasini R, Wieckowski MR, Campo G, Pinton P (2015). Molecular identity of the mitochondrial permeability transition pore and its role in ischemia-reperfusion injury. J Mol Cell Cardiol.

[68] Murphy E, Pan X, Nguyen T, Liu J, Holmström KM, Finkel T (2014). Unresolved questions from the analysis of mice lacking MCU expression. Biochem Biophys Res Commun.

[69] Neher E (1998). Vesicle pools and Ca2+ microdomains: new tools for understanding their roles in neurotransmitter release. Neuron.

[70] Nita II, Hershfinkel M, Fishman D, Ozeri E, Rutter GA, Sensi SL, Khananshvili D, Lewis EC, Sekler I (2012). The mitochondrial Na+/Ca2+ exchanger upregulates glucose dependent Ca2+ Signalling linked to insulin secretion. PLoS One.

[71] Oxenoid K, Dong Y, Cao C, Cui T, Sancak Y, Markhard AL, Grabarek Z, Kong L, Liu Z, Ouyang B, Cong Y, Mootha VK, Chou JJ (2016). Architecture of the mitochondrial calcium uniporter. Nature.

[72] Pagliarini DJ, Calvo SE, Chang B, Sheth SA, Vafai SB, Ong S-E, Walford GA, Sugiana C, Boneh A, Chen WK, Hill DE, Vidal M, Evans JG, Thorburn DR, Carr SA, Mootha VK (2008). A mitochondrial protein compendium elucidates complex I disease biology. Cell.

[73] Paillard M, Csordás G, Szanda G, Golenár T, Debattisti V, Bartok A, Wang N, Moffat C, Seifert EL, Spät A, Hajnóczky G (2017). Tissue-specific mitochondrial decoding of cytoplasmic Ca2+ signals is controlled by the stoichiometry of MICU1/2 and MCU. Cell Rep.

[74] Palty R, Silverman WF, Hershfinkel M, Caporale T, Sensi SL, Parnis J, Nolte C, Fishman D, Shoshan-Barmatz V, Herrmann S, Khananshvili D, Sekler I (2010). NCLX is an essential component of mitochondrial Na+/Ca2+ exchange. Proc Natl Acad Sci.

[75] Pan X, Liu J, Nguyen T, Liu C, Sun J, Teng Y, Fergusson MM, Rovira II, Allen M, Springer DA, Aponte AM, Gucek M, Balaban RS, Murphy E, Finkel T (2013). The physiological role of mitochondrial calcium revealed by mice lacking the mitochondrial calcium uniporter. Nat Cell Biol.

[76] Park MK, Ashby MC, Erdemli G, Petersen OH, Tepikin AV (2001). Perinuclear, perigranular and sub-plasmalemmal mitochondria have distinct functions in the regulation of cellular calcium transport. EMBO J.

[77] Patron M, Checchetto V, Raffaello A, Teardo E, Vecellio Reane D, Mantoan M, Granatiero V, Szabò I, De Stefani D, Rizzuto R (2014). MICU1 and MICU2 finely tune the mitochondrial Ca2+ uniporter by exerting opposite effects on MCU activity. Mol Cell.

[78] Paupe V, Prudent J, Dassa EP, Rendon OZ, Shoubridge EA (2015). CCDC90A (MCUR1) is a cytochrome c oxidase assembly factor and not a regulator of the mitochondrial calcium uniporter. Cell Metab.

[79] Pchitskaya E, Popugaeva E, Bezprozvanny I (2017) Calcium signaling and molecular mechanisms underlying neurodegenerative diseases. Cell Calcium. 10.1016/j.ceca.2017.06.008 10.1016/j.ceca.2017.06.008 PMC5748019 28728834

[80] Perocchi F, Gohil VM, Girgis HS, Bao XR, McCombs JE, Palmer AE, Mootha VK (2010). MICU1 encodes a mitochondrial EF hand protein required for Ca(2+) uptake. Nature.

[81] Petronilli V, Cola C, Bernardi P (1993). Modulation of the mitochondrial cyclosporin A-sensitive permeability transition pore. II. The minimal requirements for pore induction underscore a key role for transmembrane electrical potential, matrix pH, and matrix Ca2+. J Biol Chem.

[82] Petrungaro C, Zimmermann KM, Küttner V, Fischer M, Dengjel J, Bogeski I, Riemer J (2015). The Ca(2+)-dependent release of the Mia40-induced MICU1-MICU2 dimer from MCU regulates mitochondrial Ca(2+) uptake. Cell Metab.

[83] Plovanich M, Bogorad RL, Sancak Y, Kamer KJ, Strittmatter L, Li AA, Girgis HS, Kuchimanchi S, De Groot J, Speciner L, Taneja N, Oshea J, Koteliansky V, Mootha VK (2013). MICU2, a paralog of MICU1, resides within the mitochondrial uniporter complex to regulate calcium handling. PLoS One.

[84] Porporato PE, Payen VL, Pérez-Escuredo J, De Saedeleer CJ, Danhier P, Copetti T, Dhup S, Tardy M, Vazeille T, Bouzin C, Feron O, Michiels C, Gallez B, Sonveaux P (2014). A mitochondrial switch promotes tumor metastasis. Cell Rep.

[85] Qiu J, Tan Y-W, Hagenston AM, Martel M-A, Kneisel N, Skehel PA, Wyllie DJA, Bading H, Hardingham GE (2013). Mitochondrial calcium uniporter Mcu controls excitotoxicity and is transcriptionally repressed by neuroprotective nuclear calcium signals. Nat Commun.

[86] Quan X, Nguyen TT, Choi S-K, Xu S, Das R, Cha S-K, Kim N, Han J, Wiederkehr A, Wollheim CB, Park K-S (2015). Essential role of mitochondrial Ca2+ uniporter in the generation of mitochondrial pH gradient and metabolism-secretion coupling in insulin-releasing cells. J Biol Chem.

[87] Raffaello A, De Stefani D, Sabbadin D, Teardo E, Merli G, Picard A, Checchetto V, Moro S, Szabò I, Rizzuto R (2013). The mitochondrial calcium uniporter is a multimer that can include a dominant-negative pore-forming subunit. EMBO J.

[88] Rangaraju V, Calloway N, Ryan TA (2014). Activity-driven local ATP synthesis is required for synaptic function. Cell.

[89] Rasmussen TP, Wu Y, Joiner MA, Koval OM, Wilson NR, Luczak ED, Wang Q, Chen B, Gao Z, Zhu Z, Wagner BA, Soto J, McCormick ML, Kutschke W, Weiss RM, Yu L, Boudreau RL, Abel ED, Zhan F, Spitz DR, Buettner GR, Song L-S, Zingman LV, Anderson ME (2015). Inhibition of MCU forces extramitochondrial adaptations governing physiological and pathological stress responses in heart. Proc Natl Acad Sci U S A.

[90] Ringer S (1883). A third contribution regarding the influence of the inorganic constituents of the blood on the ventricular contraction. J Physiol.

[91] Rizzuto R, Simpson AW, Brini M, Pozzan T (1992). Rapid changes of mitochondrial Ca2+ revealed by specifically targeted recombinant aequorin. Nature.

[92] Rizzuto R, Brini M, Murgia M, Pozzan T (1993). Microdomains with high Ca2+ close to IP3-sensitive channels that are sensed by neighboring mitochondria. Science.

[93] Rizzuto R, De Stefani D, Raffaello A, Mammucari C (2012). Mitochondria as sensors and regulators of calcium signalling. Nat Rev Mol Cell Biol.

[94] Rottenberg H, Scarpa A (1974). Calcium uptake and membrane potential in mitochondria. Biochemistry.

[95] Rudolf R (2004). In vivo monitoring of Ca2+ uptake into mitochondria of mouse skeletal muscle during contraction. J Cell Biol.

[96] Rutter GA, Tsuboi T, Ravier MA (2006). Ca2+ microdomains and the control of insulin secretion. Cell Calcium.

[97] Rutter GA, Hodson DJ, Chabosseau P, Haythorne E, Pullen TJ, Leclerc I (2017). Local and regional control of calcium dynamics in the pancreatic islet. Diabetes Obes Metab.

[98] Sancak Y, Markhard AL, Kitami T, Kovács-Bogdán E, Kamer KJ, Udeshi ND, Carr SA, Chaudhuri D, Clapham DE, Li AA, Calvo SE, Goldberger O, Mootha VK (2013). EMRE is an essential component of the mitochondrial calcium uniporter complex. Science.

[99] Sandow A (1952). Excitation-contraction coupling in muscular response. Yale J Biol Med.

[100] Schiaffino S, Reggiani C (2011). Fiber types in mammalian skeletal muscles. Physiol Rev.

[101] Sekler I (2015). Standing of giants shoulders the story of the mitochondrial Na+Ca2+ exchanger. Biochem Biophys Res Commun.

[102] Sheng Z-H, Cai Q (2012). Mitochondrial transport in neurons: impact on synaptic homeostasis and neurodegeneration. Nat Rev Neurosci.

[103] Stumvoll M, Goldstein BJ, van Haeften TW (2005). Type 2 diabetes: principles of pathogenesis and therapy. Lancet.

[104] Tarasov AI, Semplici F, Ravier MA, Bellomo EA, Pullen TJ, Gilon P, Sekler I, Rizzuto R, Rutter GA (2012). The mitochondrial Ca2+ uniporter MCU is essential for glucose-induced ATP increases in pancreatic β-cells. PLoS One.

[105] Tarasov AI, Semplici F, Li D, Rizzuto R, Ravier MA, Gilon P, Rutter GA (2013). Frequency-dependent mitochondrial Ca(2+) accumulation regulates ATP synthesis in pancreatic β cells. Pflugers Arch.

[106] Tosatto A, Sommaggio R, Kummerow C, Bentham RB, Blacker TS, Berecz T, Duchen MR, Rosato A, Bogeski I, Szabadkai G, Rizzuto R, Mammucari C (2016). The mitochondrial calcium uniporter regulates breast cancer progression via HIF-1α. EMBO Mol Med.

[107] Toth AB, Shum AK, Prakriya M (2016). Regulation of neurogenesis by calcium signaling. Cell Calcium.

[108] Tretter L, Takacs K, Kövér K, Adam-Vizi V (2007). Stimulation of H(2)O(2) generation by calcium in brain mitochondria respiring on alpha-glycerophosphate. J Neurosci Res.

[109] Vais H, Mallilankaraman K, Mak D-OD, Hoff H, Payne R, Tanis JE, Foskett JK (2016). EMRE is a matrix Ca2+ sensor that governs gatekeeping of the mitochondrial Ca2+ uniporter. Cell Rep.

[110] Vasington FD, Murphy JV (1962). Ca ion uptake by rat kidney mitochondria and its dependence on respiration and phosphorylation. J Biol Chem.

[111] Vecellio Reane D, Vallese F, Checchetto V, Acquasaliente L, Butera G, De Filippis V, Szabò I, Zanotti G, Rizzuto R, Raffaello A (2016). A MICU1 splice variant confers high sensitivity to the mitochondrial Ca2+ uptake machinery of skeletal muscle. Mol Cell.

[112] Viola HM, Hool LC (2014). How does calcium regulate mitochondrial energetics in the heart?—new insights. Heart Lung Circ.

[113] Waldeck-Weiermair M, Malli R, Parichatikanond W, Gottschalk B, Madreiter-Sokolowski CT, Klec C, Rost R, Graier WF (2015). Rearrangement of MICU1 multimers for activation of MCU is solely controlled by cytosolic Ca(2). Sci Rep.

[114] Walkinshaw E, Gai Y, Farkas C, Richter D, Nicholas E, Keleman K, Davis RL (2015). Identification of genes that promote or inhibit olfactory memory formation in *Drosophila*. Genetics.

[115] Wang L, Yang X, Li S, Wang Z, Liu Y, Feng J, Zhu Y, Shen Y (2014). Structural and mechanistic insights into MICU1 regulation of mitochondrial calcium uptake. Embo.

[116] Wernette ME, Ochs RS, Lardy HA (1981). Ca2+ stimulation of rat liver mitochondrial glycerophosphate dehydrogenase. J Biol Chem.

[117] Wiederkehr A, Szanda G, Akhmedov D, Mataki C, Heizmann CW, Schoonjans K, Pozzan T, Spät A, Wollheim CB (2011). Mitochondrial matrix calcium is an activating signal for hormone secretion. Cell Metab.

[118] Wiser O, Trus M, Hernández A, Renström E, Barg S, Rorsman P, Atlas D (1999). The voltage sensitive Lc-type Ca2+ channel is functionally coupled to the exocytotic machinery. Proc Natl Acad Sci U S A.

[119] Wollheim CB, Sharp GW (1981). Regulation of insulin release by calcium. Physiol Rev.

[120] Wu Y, Rasmussen TP, Koval OM, Joiner M-L, Hall DD, Chen B, Luczak ED, Wang Q, Rokita AG, Wehrens XHT, Song L-S, Anderson ME (2015). The mitochondrial uniporter controls fight or flight heart rate increases. Nat Commun.

[121] Yamamoto T, Yamagoshi R, Harada K, Kawano M, Minami N, Ido Y, Kuwahara K, Fujita A, Ozono M, Watanabe A, Yamada A, Terada H, Shinohara Y (2016). Analysis of the structure and function of EMRE in a yeast expression system. Biochim Biophys Acta.

